# Randomized, double-blind trial of preoperative pregabalin versus placebo to improve quality of recovery after breast cancer surgery^☆^

**DOI:** 10.1016/j.bjane.2026.844749

**Published:** 2026-03-26

**Authors:** Fabio Vieira Toledo, Jose Fernando Amaral Meletti, Luiza Mansur Cerioni Silva, Nathalia Maria Medeiros Serra, Maria Nathalia Prado Simoes Mendonça, Paulo Henrique Carvalho Guerra, Clóvis Antônio Lopes Pinto

**Affiliations:** Escola de Medicina Jundiaí (FMJ), Department of Anesthesiology, Jundiaí, SP, Brazil

**Keywords:** Anesthesia and analgesia, Anesthesia recovery periods, Mastectomy, Postoperative pain, Pregabalin

## Abstract

**Introduction:**

Surgery remains one of the most important treatments for breast cancer. In this context, the quality of postoperative recovery has become a key concern. Adequate control of acute pain is essential to optimize patient comfort and recovery. Pregabalin may contribute to this goal by preventing central sensitization and reducing perioperative anxiety.

**Objectives:**

To evaluate the effect of perioperative pregabalin versus placebo on postoperative recovery quality in patients undergoing breast cancer surgery.

**Method:**

In this randomized controlled trial, 92 patients received either pregabalin (150 mg orally, 1 hour before surgery) or a matching placebo, both prepared in identical capsules. The primary outcome was the QoR-15 score measured preoperatively, and at 24 and 48 hours postoperatively. The 24- and 48-hour assessments were conducted via telephone. The QoR-15 is a validated instrument that assesses the quality of recovery, with total scores ranging from 0 (very poor recovery) to 150 (excellent recovery). Secondary outcomes included opioid consumption, pain scores, incidence of nausea and vomiting, and lengths of stay in the Post-Anesthesia Care Unit (PACU) and hospital. An exploratory analysis of longitudinal changes in QoR-15 scores within each group was also performed. Analyses were performed per protocol.

**Results:**

Eighty-four patients completed the study. There were no differences in overall QoR-15 score between the groups at any of the three assessment time points (preoperative, 24h, and 48h). In the exploratory longitudinal within-group analysis, better maintenance of recovery quality was observed in the pregabalin group compared with baseline, with medians (IQR) of 138 (122.3–145), 132.5 (125.8–135.3), and 134 (131.5–136) [p = 0.006 between 24h and 48h]. In the placebo group, the medians (IQR) were 140 (128–145.3), 129 (126–134.3), and 134 (126.8–136.3) [p = 0.002 between pre and 24h; p = 0.026 between pre and 48h].

**Conclusion:**

Although exploratory analysis showed a trend toward improvement within the pregabalin group, there was no significant difference in QoR-15 scores between groups.

## Introduction

Breast cancer affects millions of women worldwide. In Brazil alone, 73.610 new cases were reported in 2023.^1^ Surgery is widely recognized as one of the most important and frequently used treatments for breast cancer, particularly in cases diagnosed at early stages.^2^, ^3^, ^4^ Consequently, the management of acute postoperative pain represents a fundamental component of care, as patients often experience a combination of nociceptive and neuropathic pain. In addition, preoperative anxiety influences the subjective perception of pain and may contribute to increased postoperative pain, morbidity, and mortality.^5^ In this context, pregabalin has received attention due to emerging evidence supporting its benefit in the multimodal perioperative management of acute pain and anxiety.^6^ Despite its limited formal indications, the off-label use of pregabalin has increased substantially, making it the tenth most prescribed medication in the United States in 2017.^7^

Therefore, it is essential to assess the effectiveness of such medications using instruments that capture a patient-centered perception of recovery.

Few studies in the literature have evaluated pregabalin’s impact on the quality of recovery as a primary outcome, and none of them have specifically focused on breast cancer surgery.^8^^,^^9^ Hence, a gap remains in the literature regarding a comprehensive evaluation of pregabalin use in the perioperative period, particularly in this surgical context.

We hypothesized that preoperative pregabalin, compared with placebo, would improve the perception of recovery in patients undergoing oncologic breast surgery, as assessed by the Quality of Recovery-15 (QoR-15) questionnaire. In addition, secondary outcomes such as the incidence of nausea and vomiting, opioid consumption, pain scores, and lengths of stay in the PACU and hospital were also assessed.

## Methods

### Study design and participants

This was a prospective, randomized clinical trial. Eligible patients were aged 20–65 years, classified as ASA I or II (American Society of Anesthesiologists), and scheduled for elective oncologic breast surgery at the University Hospital of the Jundiaí Medical School, São Paulo, between September 2022 and December 2023. All procedures were performed by the same surgical team. Patients initially enrolled in the study were subsequently excluded from the analysis if they had incomplete data or did not comply with the established protocols, such as failing to complete the postoperative assessment questionnaires.

Exclusion criteria included: patient refusal; altered level of consciousness or inability to communicate; any contraindication to the use of agents described in the study protocol; history of seizure disorder; current use of pregabalin or gabapentin; presence of chronic pain or current use of opioids; insulin-dependent diabetes mellitus; and renal insufficiency (estimated glomerular filtration rate < 60 mL/min/1.73 m^2^). Patients were also excluded after randomization if there was any protocol violation, such as the use of non-protocol medications or refusal to complete the perioperative QoR-15 questionnaire.

### Randomization

Patients were randomly assigned in a 1:1 ratio to receive either pregabalin or placebo by a researcher not involved in anesthesia management or questionnaire collection. Group allocation was determined on the morning of surgery using a computer-generated random sequence (www.random.org). The study medications were prepared in identical capsules to ensure blinding. For each patient, an opaque envelope containing a single capsule (150 mg pregabalin or placebo) was prepared, sealed, and labeled with an identification number (1 or 2) by an anesthesiologist who was not involved in the study. The envelopes were stored securely by a research assistant.

One hour before the start of surgery, the assigned capsule was administered orally by a member of the clinical staff who was not involved in anesthesia care. The randomization sequence was kept confidential by one investigator until completion of data analysis. The statistician, independent from the anesthesia team and data collection, conducted all analyses while blinded to group allocation. Patients, surgeons, anesthesiologists, and all personnel involved in anesthesia management or data collection were unaware of treatment assignment.

### Outcomes

The primary outcome of the study was to evaluate the effect of perioperative pregabalin versus placebo on postoperative quality of recovery through the QoR-15 questionnaire. Additionally, an exploratory within-group (longitudinal) analysis was conducted to assess potential trends in recovery over time within each group.

Secondary outcomes included postoperative pain (at rest and during movement), opioid consumption, incidence of nausea and vomiting, length of stay in the PACU, and overall hospital length of stay.

### Anesthesia

Patients were monitored according to standard ASA guidelines. General anesthesia was induced with fentanyl (4 µg·kg^-1^), propofol (2.0 mg·kg^-1^), rocuronium (0.6 mg·kg^-1^), and esketamine (0.3 mg·kg^-1^). Sevoflurane was used to maintain the desired anesthetic depth.

Intraoperative medications included dexamethasone (10 mg), ketoprofen (100 mg), dipyrone (2000 mg), and ondansetron (4 mg). At the end of the procedure, the surgical wound was infiltrated with local anesthetic (ropivacaine 0.75%, 20 mL). Neuromuscular blockade was reversed based on Train-Of-Four (TOF) monitoring, with atropine (0.01 mg·kg^-1^) and neostigmine (20–70 µg·kg^-1^) administered according to the degree of residual blockade.

### Measurements and treatment of postoperative pain

Pain was assessed in the PACU every 15 minutes using a Verbal Numeric Rating Scale (VNRS) from 0 to 10, both at rest and during movement, where 0 represented no pain and 10 represented the worst possible pain. To assess pain during movement, patients were asked to perform a 90° arm abduction on the surgical side.

Intravenous morphine (1–2 mg) was administered every 10 minutes until a pain score below 4 was achieved (1 mg if pain < 7, 2 mg if pain ≥ 7). The occurrence of nausea and vomiting, as well as the number of vomiting episodes, was recorded. These symptoms were treated with intravenous dimenhydrinate (30 mg), and if needed, metoclopramide (10 mg) was administered subsequently.

After PACU discharge (Aldrete score ≥ 9), patients received ketoprofen 100 mg every 12 hours and oral dipyrone 500 mg every 6 hours. In the hospital ward, intravenous tramadol 100 mg was administered if the pain score exceeded 4.

Post-discharge follow-up was conducted by telephone at 24 and 48 hours, during which resting, and movement-related pain were recorded.

### Assessment of patient’s characteristics and perioperative data

Data collected included age, sex, ASA physical status, BMI, creatinine clearance (Cockcroft-Gault), duration of surgery, pain scores, and total consumption of morphine and tramadol.

The QoR-15 questionnaire was administered at three time points: 1-hour before surgery (in person) and at 24 and 48 hours postoperatively (by telephone). The QoR-15 is a short version of the QoR-40 and has been adapted into Portuguese following established guidelines (Fig. S1).^10^

It consists of 15 items divided into two parts: A and B. In Part A, the items reflect positive aspects of recovery and are rated on an 11-point numerical scale ranging from 0 (“none of the time”) to 10 (“all of the time”). In Part B, the scoring is reversed: 10 corresponds to “none of the time” and 0 to “all of the time”.

The QoR-15 evaluates five key dimensions of recovery: pain (2 items), physical comfort (5 items), physical independence (2 items), psychological support (2 items), and emotional state (4 items). The total score ranges from 0 (poor recovery) to 150 (excellent recovery).

### Sample size

A sample size of 37 patients per group was estimated to achieve 85% power to detect a 12-point difference in the QoR-15 score, with a standard deviation of 17. This calculation was based on a 12-point difference in mean scores observed in a previous study using the QoR-15 in medium- and major-sized surgeries, as this reflects a realistic effect expected in our population. The Standard Deviation (SD) of QoR-15 scores in that study ranged from 17 to 18; therefore, an SD of 17 was considered acceptable.^11^ Considering potential dropouts during the study, a total of 92 patients were recruited.

### Ethics statement

This study was approved by the University Hospital of Jundiaí and the Ethics Committee (CAAE: 60819922.0.0000.5412, approved on 20/09/2022) at the Jundiaí Medical, registered on the Brazilian Clinical Trials Registry website (REBEC ‒ U1111-1278-3266, approved on 14/03/2023), and all patients were informed and signed the Informed Consent Form.

### Statistical analysis

After data collection, the distribution of the primary outcome was assessed using the Kolmogorov-Smirnov test and did not meet the assumption of normality; therefore, it was expressed as median and Interquartile Range (IQR). The groups (independent samples) were compared using the Mann-Whitney test for all quantitative factors. Categorical data and their frequencies were compared using the χ² test. Finally, for the exploratory analysis of QoR-15 evolution in each group (preoperative, 24 and 48 hours), since there were three measurement time points, the Friedman test was used, followed by the Wilcoxon test for pairwise post-hoc comparisons. Additionally, a subgroup analysis was performed excluding minor surgical procedures to assess whether surgery size influenced the results for both the primary outcome and pain scores.

The criterion for rejecting the null hypothesis was a p < 0.05 for both primary and secondary outcomes. Statistical analyses were performed using SPSS V26 (2019), Minitab 21.2 (2022), and Excel Office 2010.

## Results

**Figure 1:**
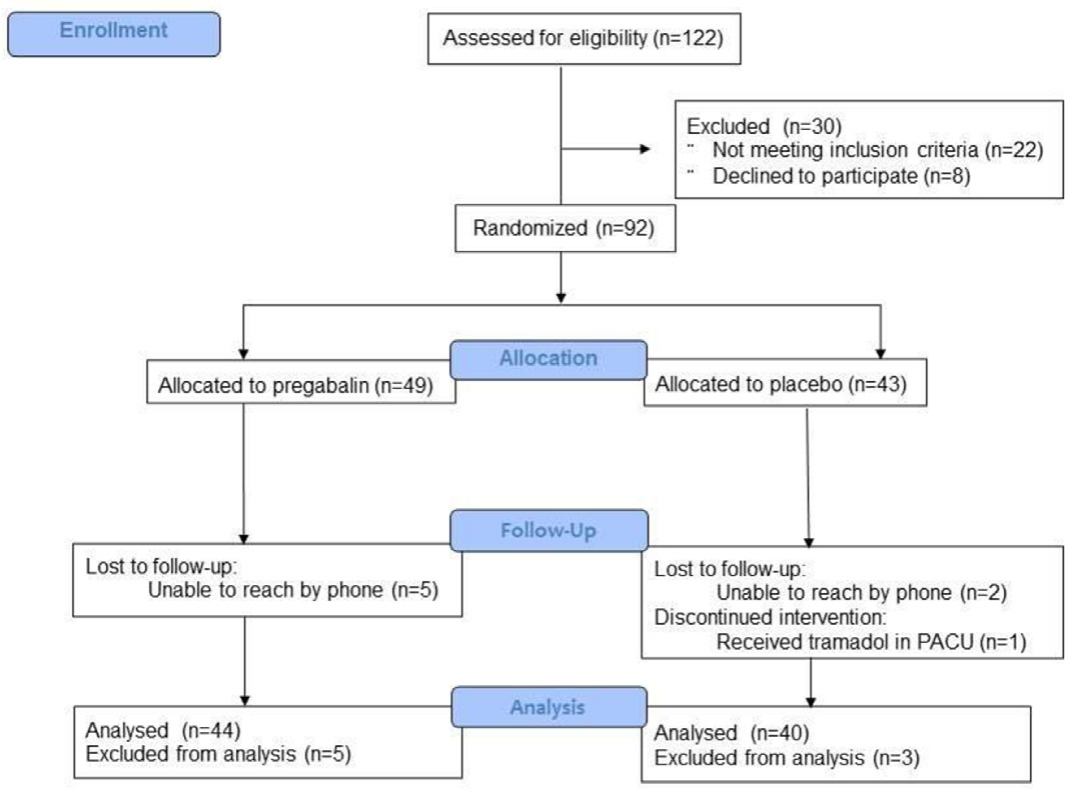
CONSORT Flow diagram of patient enrollment in the study.

Initially, 92 patients were enrolled in the trial; however, 8 were excluded after randomization: 7 due to failure to reach patients by phone to complete the 24- and 48-hour questionnaires, and 1 for receiving a dose of tramadol in the PACU, which was not part of the study’s analgesic regimen. Following the per-protocol analysis, 84 patients were included in the final analysis, with 44 in the pregabalin group and 40 in the placebo group (Fig. 1).

It was noted that both groups consisted mainly of patients classified as ASA 2. The most common surgery performed was segmental mastectomy without axillary dissection, resulting in a predominance of medium-sized surgeries in both groups. Four complications were observed, all of which were minor, occurring during the intraoperative and immediate postoperative periods: one intraoperative hypertensive peak in each group, one episode of delirium upon awakening in the placebo group, and one vascular injury related to the surgical procedure in the pregabalin group. There were no statistically significant differences in demographic or clinical characteristics between the groups (Table 1 and S1).

**Table 1:** Demographic data and patient characteristics.

Variables	Placebo (n = 40)	Pregabalin (n = 44)	p-value
Age (years), Median (Q_1_ ‒ Q_3_)	49 (43.3 – 57.5)	53 (44.7 – 59)	0.754^a^
Height (m), Median (Q_1_ ‒ Q_3_)	1.6 (1.5 – 1.6)	1.6 (1.5 – 1.6)	0.896^a^
Weight (kg), Median (Q_1_ ‒ Q_3_)	71 (62.8 – 83.3)	71.5 (64 – 78.5)	0.657^a^
BMI (kg.m^-2^), Median (Q_1_ ‒ Q_3_)	28.4 (25 – 31.5)	27.5 (25.1 – 30.2)	0.658^a^
ASA I:II, n	6:34	6:38	0.858^b^
Previous surgery, n (%)	28 (70%)	30 (68.2%)	0.857^b^
Surgical duration (min), Median (Q_1_ ‒ Q_3_)	57.5 (40 – 75)	50 (40 – 60)	0.142^a^
Time to awakening (min), Median (Q_1_ ‒ Q_3_)	12.5 (10 – 15)	10 (10 – 15)	0.732^a^
Surgery complexity, n (%)^c^			
Major	4 (10%)	6 (13.6%)	0.607^b^
Moderate	32 (80%)	33 (75%)	0.584^b^
Minor	4 (10%)	5 (11.4%)	0.840^b^
Complications	2 (5%)	2 (4.5%)	0.922^b^

Q1, First Quartile; Q3, Third Quartile.

a Mann-Whitney test.

b χ² test.

c Major, including unilateral or bilateral mastectomy, with or without prosthesis removal or placement; Moderate, segmental mastectomy or wide local excision; Minor, Unilateral excisional biopsy and nodulectomy.

**Figure 2:**
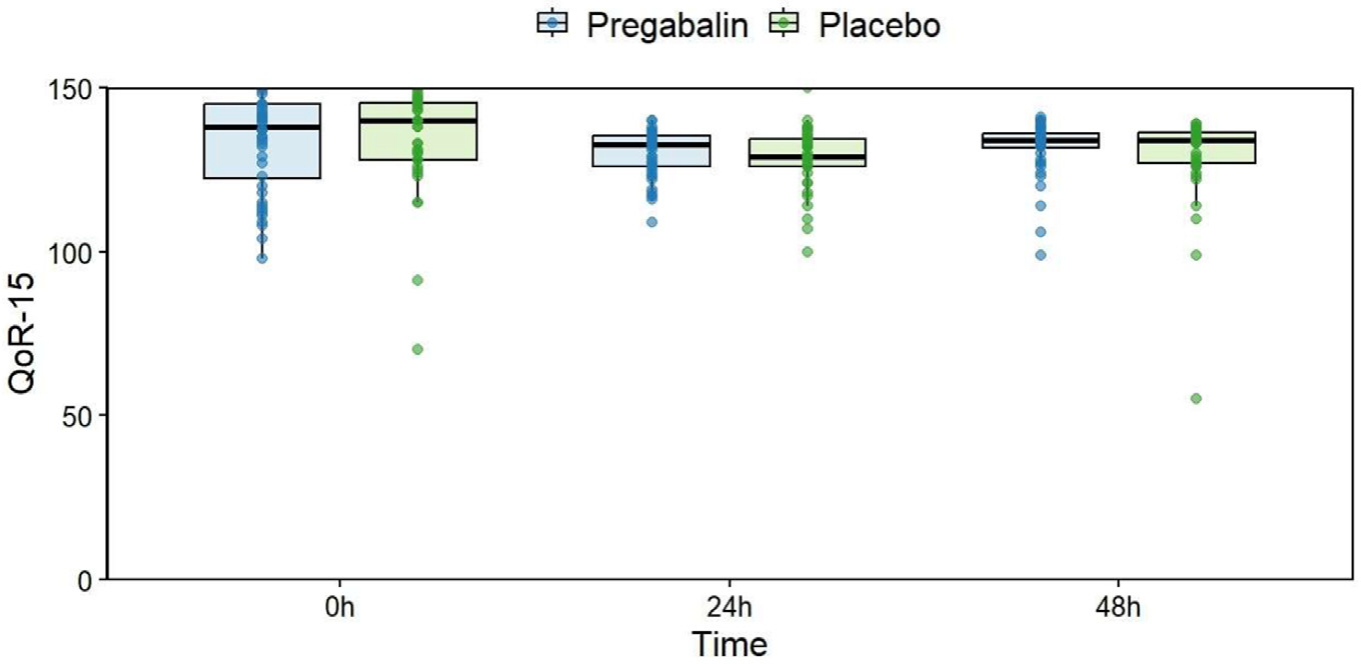
Comparison of QoR-15 scores between groups. Data are presented as median (interquartile range); points represent individual patients.

A comparison of the QoR-15 scores at three time points (preoperative, 24 hours, and 48 hours) revealed no significant difference between the groups (Table 2 and Fig. 2).

**Table 2:** QoR-15 scores at the three time points in each group.

	**Time points**	**Placebo**	**Pregabalin**	**p-value**^b^
**QoR-15**^a^	Pre	140 (128 ‒ 145.3)	138 (122.3 – 145)	0.616
	24 hours	129 (126 ‒ 134.3)	132.5 (125.8 – 135.3)	0.495
	48 hours	134 (126.8 ‒ 136.3)	134 (131.5 – 136)	0.634

a Median and Interquartile Range.

b Mann-Whitney test.

Next, an exploratory longitudinal analysis of the QoR-15 was performed to evaluate changes in scores within each group. The three time points were first assessed, followed by pairwise post-hoc comparisons between assessment points. The p-values from Table 3 indicate a statistical difference in the evolution of the QoR-15 score at different time points in both groups, with a p = 0.002 for the placebo group and a p = 0.008 for the pregabalin group. In the placebo group, the median started at 140 preoperatively, dropped to 129 at 24 hours, and then increased to 134 at 48 hours. Post-hoc analysis revealed a significant decrease in the QoR-15 score between the preoperative time and both postoperative time points (p = 0.002 for 24 hours and p = 0.026 for 48 hours). In the pregabalin group, the median started at 138, dropped to 132.5 at 24 hours, and ended at 134 at 48 hours. Post-hoc analysis showed only a significant difference between the 24-hour and 48-hour postoperative scores (p = 0.006), representing an improvement in the QoR-15 median during this interval (Table 3).

**Table 3:** Compares the evolution of QoR-15 score by Group and post-hoc p-values.

	QoR Pre	QoR 24 hours	QoR 48 hours	p-value			
				Total^b^	Pre/24^c^	Pre/48^c^	24/48^c^
**Placebo**^a^	140 (128 ‒ 145.3)	129 (126 ‒ 134.3)	134 (126.8 ‒ 136.3)	0.002	0.002	0.026	0.054
**Pregabalin**^a^	138 (122.3 – 145)	132.5 (125.8 – 135.3)	134 (131.5 – 136)	0.008	0.176	0.398	0.006

a Median and Interquartile Range.

b Friedman test.

c Wilcoxon test.

**Figure 3:**
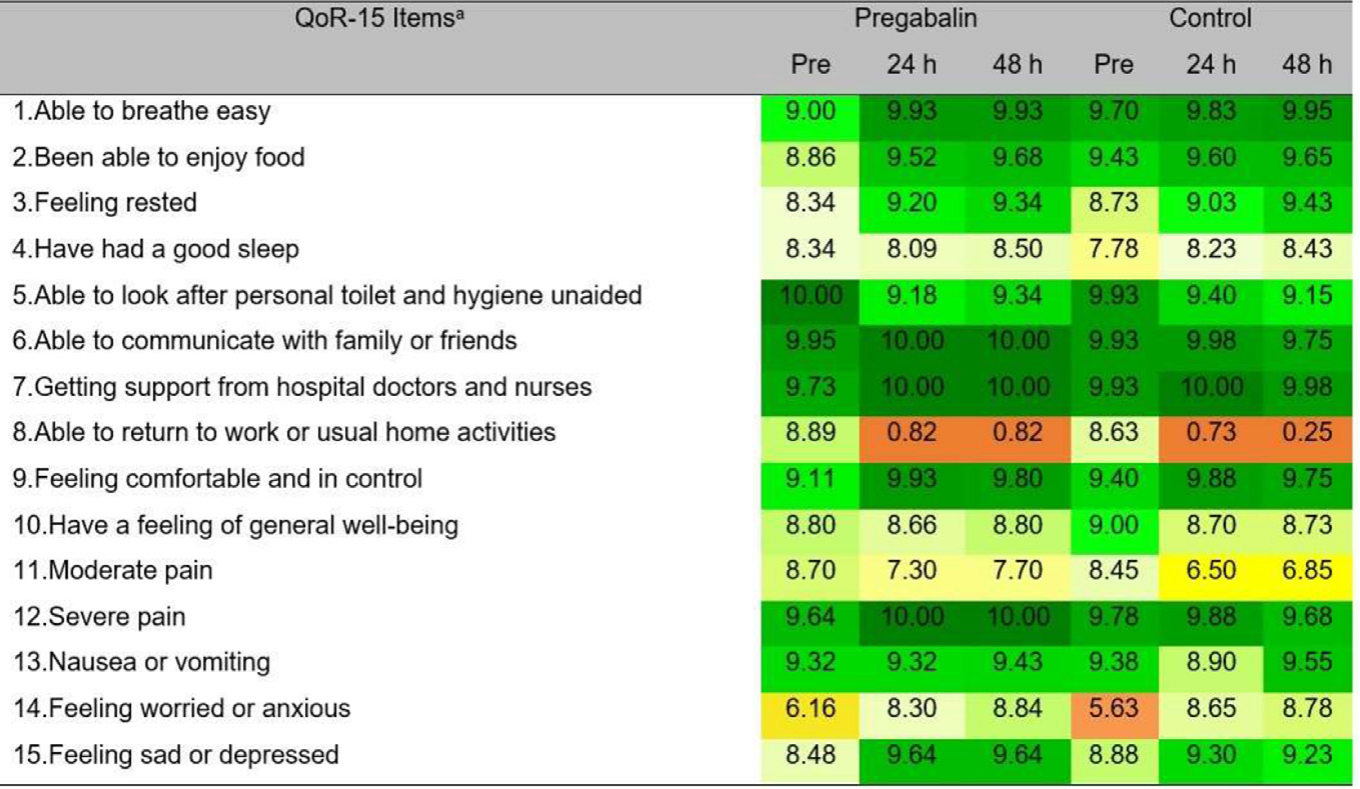
Heat map of the mean score for each QoR-15 item at the three perioperative time points. Part A (questions 1–10) and Part B (questions 11–15). Colors indicate score levels: dark green = high, yellow = medium, orange = low. Higher scores reflect better recovery. ^a^ Mean scores for each item.

Analyzing Figure 3, which shows the heatmap of average QoR-15 item scores, it can be observed that “severe pain” was not a concern in the postoperative period for either group. The item with the lowest score in the preoperative period was the question “I felt worried or anxious”, and in the postoperative period, the lowest scores were for “moderate pain” and “ability to return to work or domestic activities”.

Postoperative pain scores, assessed using the Verbal Numerical Rating Scale (VNRS), did not differ between groups in the PACU, at 24 hours, or at 48 hours after surgery. Pain levels were low, with most median values in the PACU being 0 and interquartile ranges also at 0 (Table S2).

No significant differences were observed between the groups in the incidence of nausea and vomiting in the PACU and hospital ward, morphine consumption, or length of stay in the PACU and hospital (Table S3).

A subgroup analysis was performed, excluding minor surgeries (unilateral excisional biopsy and nodulectomy), evaluating both the primary outcome and pain. However, the null hypothesis could not be rejected, and the median values were very similar to those observed in the total study population (Table S4).

## Discussion

The primary outcome of this study did not reveal significant differences in total QoR-15 scores between the pregabalin and placebo groups at 24 and 48 hours postoperatively. Similarly, no differences were observed in pain scores or opioid consumption. Exploratory longitudinal analyses within each group suggest that preoperative oral pregabalin may help maintain recovery quality over the postoperative period; however, these findings are hypothesis-generating and require confirmation using pre-specified longitudinal models in future studies.

Currently, both gabapentin and pregabalin are used to enhance postoperative recovery, serving as multimodal medication strategies for the control of acute pain and perioperative anxiety.^5^^,^^12^, ^13^, ^14^, ^15^, ^16^ Several studies have been conducted evaluating pain and opioid consumption, and a recent meta-analysis demonstrated that pregabalin has modest efficacy in managing acute pain after breast cancer surgery, with greater efficiency in reducing chronic postoperative pain after 3 months. Acute pain at rest 24 hours after surgery was reduced by 0.31 points on a 0–10 VNRS (95% CI 0.05 to 0.57; p = 0.02).^17^

As demonstrated by Verret et al., it is crucial to consider a minimum difference of 10% on the pain scale to support the evidence that there is a clinically significant analgesic effect. Their meta-analysis found no relevant effects of gabapentinoids on postoperative pain ‒ acute, subacute, or chronic ‒ were found. This analysis included a wide range of surgical procedures, from endoscopic to major surgeries, with high heterogeneity, highlighting the variability of the included studies.^18^^,^^19^

It is known that determining whether a medication is effective or not depends on many factors, including the type of surgery performed and its extent. Mastectomies, in particular, are surgeries that involve nociceptive mechanisms, especially when the axillary region is involved.^15^ Other studies have evaluated gabapentin specifically in oncologic breast surgery. For example, Jiang et al. demonstrated a reduction of 16.14 points (95% CI: 10.24–21.85; p < 0.001) and 27.33 points (95% CI: 3.63–51.03; p = 0.024) on the 110-point Visual Analog Scale (VAS) immediately after surgery and at 24 hours, respectively.^20^

Rai and colleagues also conducted a meta-analysis in the context of oncologic breast surgery, expanding the evaluation to include not only gabapentin but also pregabalin. A total of 516 patients were included in the gabapentin group and 209 in the pregabalin group. In the gabapentin group, using the 0–10 VNRS, pain in the PACU was reduced by 1.68 points (95% CI 0.77–2.59; p < 0.001), by 0.52 points (95% CI: 0.01–1.02; p = 0.04) at 24 hours, and morphine consumption on the first postoperative day decreased by 4.00 mg (95% CI 0.91–7.09; p = 0.01).^15^

In the same meta-analysis, only four pregabalin studies were included, and acute pain scores were reported in two trials. Pain in the PACU was reported in a single trial, showing a significant reduction in the pregabalin group (p = 0.01), while pain at 24 hours postoperatively was reported in both trials, with no significant reduction observed when pooled (p = 0.21). Three studies reported morphine consumption in the PACU, demonstrating a significant reduction of 4.8 mg (95% CI 0.83‒8.76; p = 0.02) in the pregabalin group. It is noteworthy that two studies (accounting for nearly 60% of the patients in this subgroup) used a 75 mg pregabalin dose, one used 150 mg (16%), and the highest dose used was 300 mg (24%).^15^ The use of multiple dosing regimens, in addition to the limited number of studies included in the analysis, may be the main reasons for the conflicting results reported in the literature.

However, the efficacy of a multimodal analgesic approach should not be assessed solely based on pain scores, as effective pain control methods with morphine or gabapentinoids may not reflect differences in final pain scores. In addition, postoperative opioid consumption is not considered a patient-centered outcome, as it may reflect both improved analgesia and decreased comfort or the occurrence of adverse events.^21^ In this way, there is currently a growing concern regarding patients’ perception and evaluation of the healthcare they receive. This perspective is often summarized by the expression “Through the Patient’s Eyes”.^22^

Borde et al. conducted a randomized clinical trial in patients undergoing off-pump coronary artery bypass surgery. Participants received 150 mg of pregabalin preoperatively, followed by 75 mg twice daily for 2 days after extubation. The primary outcome was the quality of recovery, assessed using the QoR-40 questionnaire. Patients in the pregabalin group had higher global QoR-40 scores 24 hours after extubation compared with the placebo group (177 ± 9 vs. 170 ± 9; p = 0.002). The pregabalin group also showed better scores in the emotional state, physical comfort, and pain dimensions.^9^ Martins et al. also conducted an investigation in individuals undergoing bariatric surgery (non-laparoscopic); however, they did not find an improvement in recovery quality (assessed by QoR-40), raising the question of whether the lack of effect was due to the low pregabalin dose of 75 mg.^8^

Hetta et al. conducted a clinical trial in the context of oncologic breast surgery, aimed at predicting the optimal dose for achieving analgesic effects with minimal adverse effects. They compared placebo with three escalating doses of pregabalin (75, 150, and 300 mg) and found that a single preoperative dose of 150 mg pregabalin reduced pain and morphine consumption in the first 24 hours after mastectomies without increasing the incidence of pregabalin-related adverse events such as dizziness or visual disturbances.^23^

Based on these findings, our study selected a single preoperative dose of 150 mg pregabalin. However, we did not find statistically significant differences in pain scores (PACU, 24 and 48 hours) or opioid consumption. This result can partly be attributed to the multimodal analgesia employed both in the anesthetic technique and in the postoperative period for both groups (dexamethasone, ketoprofen, dipyrone, esketamine, local infiltration, and morphine rescue), which may have led to a redundant effect of pregabalin in this context of optimized multimodal analgesia.

Previous studies have shown that other agents, such as esketamine, can improve the quality of anesthetic recovery not only through analgesic effects and attenuation of hyperalgesia but also via their antidepressant properties. For instance, Gao et al. observed that esketamine contributed to better postoperative recovery in patients undergoing mastectomies, likely due to these multifaceted effects.^24^ Considering that a significant proportion of women with breast cancer experience postoperative depression,^25^ such pharmacological properties may enhance overall recovery.

Furthermore, most of the patients underwent small and medium-sized surgeries without axillary dissection, which are associated with lower nociceptive mechanisms.^15^ To evaluate whether surgical extent could influence the results, we performed a subgroup analysis excluding minor procedures, such as nodulectomies and excisional biopsies. No differences in the primary outcome were observed at any time point between the groups. However, most of the included procedures were of moderate extent, such as segmental mastectomy without axillary dissection. It was not possible to perform an analysis exclusively on major surgeries, as the sample size for this type of procedure was small, which could compromise the validity of any conclusions.

As highlighted by Campfort et al., longitudinal analysis of individual patient trajectories can identify those with recovery below expectations, allowing more personalized perioperative management.^26^ In our study, longitudinal analysis showed that pregabalin group-maintained baseline recovery scores in the first 24 hours, with an increase from 24 to 48 hours postoperatively. Conversely, the placebo group experienced a decline in recovery quality at 24 hours, which persisted at 48 hours. It was observed that the median in the placebo group decreased by 11 points 24 hours after surgery, which, according to Myles et al., represents a clinically relevant change of 8 points on the scale.^11^

This finding raises concern, as a reduced score within the first 24 hours after surgery is correlated with complications that can persist up to a month after the procedure.^26^ Additionally, Chazapis et al. emphasized the importance of the 48-hour postoperative questionnaire approaching preoperative baseline levels. Failure to achieve this return may indicate undesirable recovery drift. This aspect was observed in the placebo group in our study, where the comparison of scores at 24 and 48 hours postoperatively was significantly lower than the preoperative baseline.^27^

In this study, most included procedures were segmental mastectomy without axillary dissection, generally associated with moderate complexity, lower pain levels, and faster recovery. This predominance of less extensive surgeries may limit the generalizability of our findings to mastectomies and other more extensive procedures, in which pregabalin could potentially have a greater impact. Therefore, many patients had high scores on the questionnaire, possibly reaching a ceiling effect, particularly on questions 11 and 12, which addressed pain. As a result, small differences in questionnaire scores may have gone undetected. The use of a robust multimodal analgesic regimen and local infiltration likely minimized detectable between-group differences in pain and overall QoR-15 scores. An additional limitation identified was the lack of Patient-Controlled Analgesia (PCA), which may have implications for pain perception and management in the postoperative period. Moreover, we did not assess pain during the period between PACU discharge and the first 24 hours post-procedure. We also did not systematically evaluate adverse effects such as visual disturbances or sedation using validated scales, which could have provided further insights into patient comfort and well-being. Finally, an important methodological limitation is the absence of pre-specified longitudinal modeling. Although exploratory within-group analyses in the pregabalin group suggest a more favorable recovery trajectory, these findings should be interpreted with caution. It is crucial to consider these limitations when interpreting the results and applying the conclusions of this study.

## Conclusion

A single preoperative dose of 150 mg pregabalin did not produce significant differences in total QoR-15 scores between groups at 24 and 48 hours, nor in pain control or opioid consumption. In an exploratory longitudinal analysis, within-group trajectories suggest that pregabalin may help maintain recovery quality over the postoperative period, a finding that requires confirmation using pre-specified longitudinal models.

## Declaration of competing interest

The authors declare no conflicts of interest.

## Appendix Supplementary materials

Appendices Image, application 1

## Footnotes

Supplementary material associated with this article can be found, in the online version, at doi:10.1016/j.bjane.2026.844749.
