# Supplementary material for: Randomized, double-blind trial of preoperative pregabalin versus placebo to improve quality of recovery after breast cancer surgery
Source: Braz J Anesthesiol. 2026 Mar 26;76(3):844749. doi: 10.1016/j.bjane.2026.844749 (PMC13157063; doi:10.1016/j.bjane.2026.844749)
Supplement: Supplementary file 1 — Appendices [file mmc1.docx]

**BJAN-D-25-00420**

**SUPPLEMENTARY MATERIAL 1**

**Table of Contents**

**Supplementary Figure S1 -** **QoR-15 questionnaire** (part A and B).

**Supplementary Table S1 -** Surgeries performed.

**Supplementary Table S2 -** Pain at rest and during movement.

**Supplementary Table S3 -** Additional outcomes of interest.

**Supplementary Table S4 -** Subgroup analysis excluding minor surgeries.

| Date / / |  | Study #: ____ |
| --- | --- | --- |
| Preoperative □ | 24 hours □ | 48 hours □ |
| 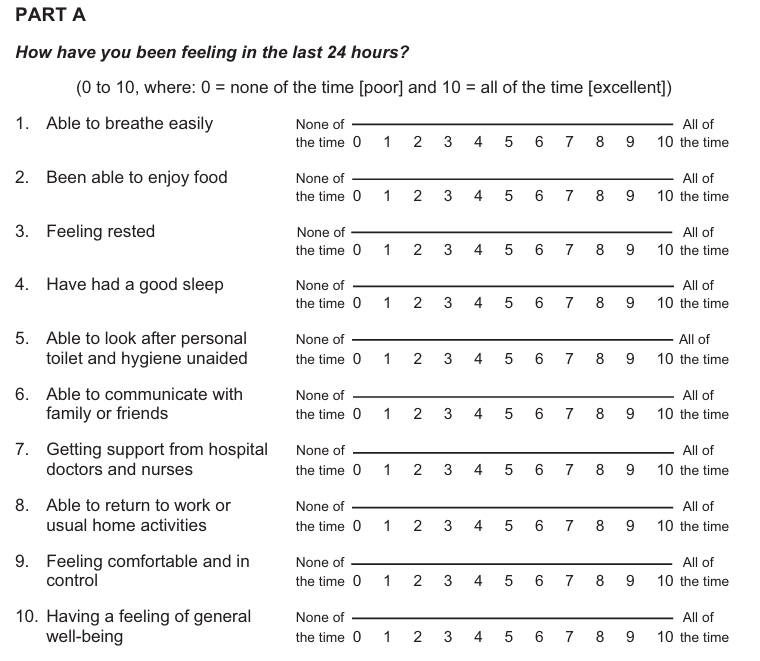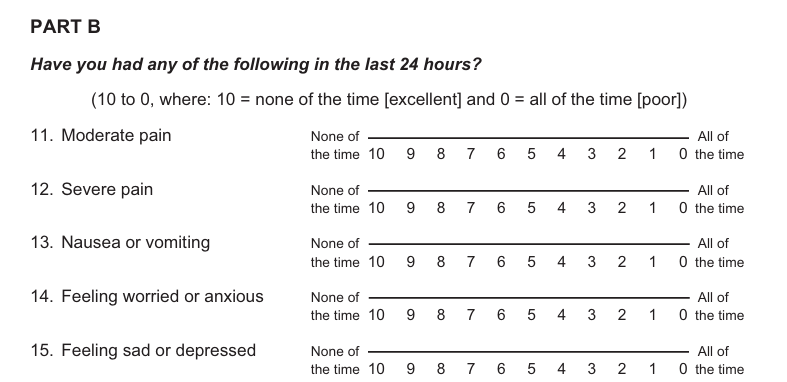 | | |

**Figure S1 –** QoR-15 patient questionnaire – Parts A and B.

**Table S1 –** Surgeries performed.

|  | **Placebo (n = 40)** | **Pregabalin**  **(n = 44)** | **P-value**^a^ | |
| --- | --- | --- | --- | --- |
| Types of surgeries performed, n (%) |  |  |  |  |
| Segmental mastectomy + SLNB | 31 (77.5%) | 30 (68.2%) | **0.339** |  |
| Segmental mastectomy + ALND | 1 (2.5%) | 0 | **0.291** |  |
| Wide local excision + SLNB | 0 | 3 (6.8%) | **0.093** |  |
| Unilateral mastectomy | 2 (5%) | 6 (13.6%) | **0.178** |  |
| Unilateral excisional biopsy | 1 (2.5%) | 2 (4.5%) | **0.614** |  |
| Nodulectomy | 3 (7.5%) | 3 (6.8%) | **0.904** |  |
| Left prosthesis removal and right mastectomy | 1 (2.5%) | 0 | **0.291** |  |
| Bilateral mastectomy and bilateral prosthesis placement | 1 (2.5%) | 0 | **0.291** |  |

^a^ χ² test.

ALND, Axillary Lymph Node Dissection; SLNB, Sentinel Lymph Node Biopsy.

**Table S2 -** Pain at rest and during movement.

|  | | **Time** | **Placebo** | **N** | **Pregabalin** | **N** | **P-value**^a^ | |
| --- | --- | --- | --- | --- | --- | --- | --- | --- |
| Pain at rest (PACU)^b^ | | 0’ | 0 (0-0) | 40 | 0 (0-0) | 44 | **0.175** | |
|  | | 15’ | 0 (0-0) | 40 | 0 (0-0) | 44 | **0.138** | |
|  | | 30’ | 0 (0-0) | 40 | 0 (0-0) | 44 | **0.360** | |
|  | | 45’ | 0 (0-0) | 38 | 0 (0-0) | 40 | **0.765** | |
|  | | 60’ | 0 (0-0) | 35 | 0 (0-0) | 36 | **0.631** | |
|  | | 75’ | 0 (0-0) | 16 | 0 (0-0) | 19 | **0.359** | |
|  | | 90’ | 0 (0-0) | 16 | 0 (0-0) | 15 | **1.000** | |
| Pain on movement (PACU)^b^ | | 0’ | 0 (0-0) | 40 | 0 (0-0) | 44 | **0.175** | |
|  | | 15’ | 0 (0-0) | 40 | 0 (0-0) | 44 | **0.196** | |
|  | | 30’ | | 0 (0-0) | 40 | 0 (0-0) | 44 | **0.220** |
|  | | 45’ | 0 (0-0) | 38 | 0 (0-0) | 40 | **0.808** | |
|  | | 60’ | 0 (0-0) | 35 | 0 (0-0) | 36 | **0.950** | |
|  | | 75’ | 0 (0-0) | 16 | 0 (0-0) | 19 | **0.359** | |
|  | | 90’ | 0 (0-0) | 16 | 0 (0-0) | 15 | **1.000** | |
| Pain at rest^b^ | | 24hs | 0 (0-2) | 40 | 0 (0-0.2) | 44 | **0.425** | |
|  | | 48hs | 0 (0-2) | 40 | 0 (0-1) | 44 | **0.846** | |
| Pain on movement^b^ | | 24hs | 6.5 (3.7-8) | 40 | 6 (2.7-8) | 44 | **0.779** | |
|  | | 48hs | 6.5 (4.5-8) | 40 | 7 (2-8) | 44 | **0.935** | |

^a^ Mann-Whitney test.

^b^ Results expressed in Median (interquartile range).

**Table S3 –** Additional outcomes of interest.

|  | **Placebo (n = 40)** | **Pregabalin**  **(n = 44)** | **P-value** |
| --- | --- | --- | --- |
| Nausea and vomiting in the PACU, n (%) | 1 (2.5%) | 2 (4.5%) | **0.614**^a^ |
| Nausea and vomiting in the ward, n (%) | 1 (2.5%) | 3 (6.8%) | **0.353**^a^ |
| Morphine consumption (mg), Median (Q_1_ – Q_3_) | 0 (0-0) | 0 (0-0) | **0.550**^b^ |
| PACU time (min), Median (Q_1_ ‒ Q_3_) | 60 (60 – 90) | 60 (60 – 90) | **0.765**^b^ |
| Hospital length of stay (hours), Median (Q_1_ ‒ Q_3_) | 17.5 (13 – 25) | 18 (14 – 24) | **0.950**^b^ |

^a^ χ² test.

^b^ Mann-Whitney test.

**Table S4 –** Subgroup analysis of pain and QoR-15 scores between groups (excluding minor surgeries).

|  | **Time** | | **Placebo** | **N** | **Pregabalin** | **N** | **P-value**^a^ | |
| --- | --- | --- | --- | --- | --- | --- | --- | --- |
| Pain at rest (PACU)^b^ | 0’ | | 0 (0-0) | 36 | 0 (0-0) | 39 | **0.171** | |
|  | 15’ | | 0 (0-0) | 36 | 0 (0-0) | 39 | **0.141** | |
|  | 30’ | | 0 (0-0) | 36 | 0 (0-0) | 39 | **0.368** | |
|  | 45’ | | 0 (0-0) | 34 | 0 (0-0) | 36 | **0.743** | |
|  | 60’ | | 0 (0-0) | 31 | 0 (0-0) | 33 | **0.603** | |
|  | 75’ | | 0 (0-0) | 14 | 0 (0-0) | 18 | **0.378** | |
|  | 90’ | | 0 (0-0) | 14 | 0 (0-0) | 14 | **1.000** | |
| Pain on movement (PACU)^b^ | 0’ | | 0 (0-0) | 36 | 0 (0-0) | 39 | **0.171** | |
|  | 15’ | | 0 (0-0) | 36 | 0 (0-0) | 39 | **0.198** | |
|  | 30’ | 0 (0-0) | 36 | 0 (0-0) | 39 | **0.226** |  |  |
|  | 45’ | | 0 (0-0) | 34 | 0 (0-0) | 36 | **0.974** | |
|  | 60’ | | 0 (0-0) | 31 | 0 (0-0) | 33 | **0.638** | |
|  | 75’ | | 0 (0-0) | 14 | 0 (0-0) | 18 | **0.378** | |
|  | 90’ | | 0 (0-0) | 14 | 0 (0-0) | 14 | **1.000** | |
| Pain at rest^b^ | 24hs | | 0 (0-2.5) | 36 | 0 (0-1) | 39 | **0.460** | |
|  | 48hs | | 0 (0-2) | 36 | 0 (0-1.5) | 39 | **0.899** | |
| Pain on movement^b^ | 24hs | | 7 (4.75-8) | 36 | 6 (3.5-8) | 39 | **0.677** | |
|  | 48hs | | 7 (5-8) | 36 | 7 (4-8) | 39 | **0.885** | |
| QoR-15^b^ | Pre  24hs  48hs | | 140 (128-145)  129.5 (126-134.2)  133.5 (126-136) | 36  36  36 | 137 (121.5-144.5)  132 (126-135)  134 (129-136) | 39  39  39 | **0.510**  **0.625**  **0.503** | |

^a^ Mann-Whitney test.

^b^ Results expressed in Median (interquartile range).
